# Adult Spinal Deformities: Can Patient-Specific Rods Change the Preoperative Planning into Clinical Reality? Feasibility Study and Preliminary Results about 77 Cases

**DOI:** 10.1155/2020/6120580

**Published:** 2020-07-10

**Authors:** S. Prost, S. Pesenti, K. Farah, P. Tropiano, S. Fuentes, B. Blondel

**Affiliations:** Aix-Marseille Université, APHM, CNRS, ISM, Hôpital de la Timone, Unité de Chirurgie Rachidienne, 264 Rue Saint Pierre, Marseille 13005, France

## Abstract

Surgical management of adult spinal deformities remains challenging, and one of the major goals is to restore sagittal alignment. Spinal rods used for posterior fixation are usually delivered straight and bended manually during surgery. This manual bending can be responsible for undercorrection of the deformity. In the last years, prebended patient-specific rods have been developed and might be a valuable tool in order to optimize surgical results. The objective is therefore to use the time between surgical decision and operative room in order to realize a precise surgical planning and obtain patient-specific rods. We describe here the planning process and our preliminary experience with patient-specific rods in the management of adult deformity about 77 cases. On the 77 cases, PSR were used without further modifications of the shape. Based on 3-month postoperative evaluation, a significant decrease of sagittal vertical axis (−41%, *p* < 0.0001) and pelvic incidence-lumbar lordosis (−62%, *p* < 0.0001) was reported. Pelvic tilt was not significantly corrected, except in patients with Parkinson's disease. In this subgroup of patients, measurements revealed a significant correction of SVA and PI-LL (−53%, *p*=0.005, and −81%, *p* < 0.0001, respectively) but also of PT (−23%, *p* < 0.001). The use of PSR, in our experience, was feasible and provided satisfactory short-term results. It can be a valuable tool in the management of adult spinal deformities. Further studies will be needed in order to confirm these preliminary results.

## 1. Introduction

Prevalence of adult spinal deformity (ASD) has been reported as high as 32% of the population and up to 68% of patients over 60 years old or patients with Parkinson's disease [1, 2]. These deformities can be responsible for various disability and increasing healthcare costs with respect to global population aging [1].

In some cases, when spinal deformity leads to high level of disability, surgical management may be warranted. If so, common strategy includes a posterior fixation and a sagittal realignment procedure (osteotomy). Data from the literature have widely reported the positive impact of sagittal realignment in terms of health-related quality of life scores [3].

Despite the development in the last decades of specific tools (rods, screws, and intervertebral cages) and surgical techniques (osteotomies), a large proportion of patients remain undercorrected after surgery [4]. One of the surgical steps that remains unchanged is related to the manual bending of spinal rods, which is probably nor precise or reproducible.

It can therefore seem logical to develop patient-specific rods (PSR) based on a preoperative surgical planning, in order to obtain rods that would provide a frame for each patient correction individually. Potential results of this strategy will be associated to a better correction of the deformity, a lower risk of rod breakage, and a reduction of operative time [5, 6].

The aim of this study was to describe the surgical planning used in order to obtain PSR and to report our preliminary experience using PSR in the management of ASD.

## 2. Methods

**Figure 1:**
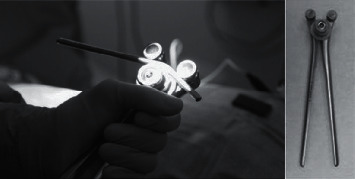
classic French bender use to shape rods.

**Figure 2:**
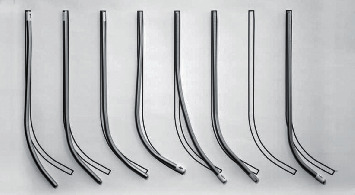
Example of rod bending by 7 surgeons showing the difficulty of reproducibility.

**Figure 3:**
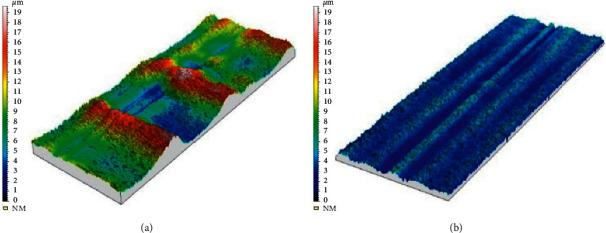
3D high-resolution surface view of titanium rods bended manually (a) and industrially (b). Notches are visible on the surface of manually bended rods.

Surgical management of ASD usually requires a posterior vertebral fixation in association to a posterior bone resection for sagittal realignment. In daily practice, the surgeon will preoperatively decide levels of fixation, type of osteotomy, need for intervertebral graft, and sagittal alignment objectives. However, so far, rod bending is performed manually during surgery (Figure 1) using straight rods provided by the manufacturer. This step is therefore not always reproducible (Figure 2) and is associated with a risk of postoperative undercorrection. It can also be theoretically with an increased risk of rod breakage due to the use of the French bender (Figure 3).

Recently, a company offered the possibility to obtain PSR based on a preoperative planning for each patient, using calibrated full-spine radiographs. The surgical planning can be done directly by the surgeon or by the company (Medicrea International, Rillieux-la-Pape, France) according to various parameters: levels of fixation, level and type of osteotomy, sagittal correction objectives, and rod material (titanium or cobalt-chrome).

**Figure 4:**
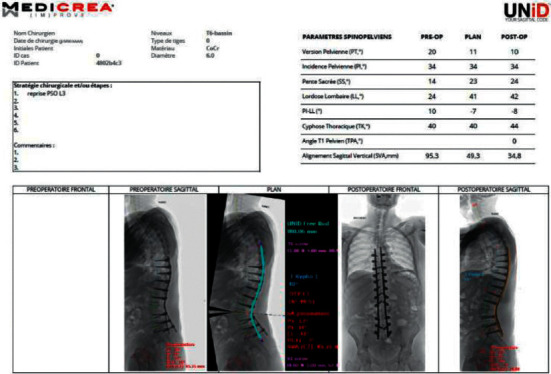
Example of surgical planning. Revision (a) with L3 pedicle subtraction osteotomy with simulation of the postoperative result, shape of the PSR, and postoperative results (b).

Using dedicated software and based on a calibrated lateral full-spine X-ray, a simulation of postoperative alignment is obtained in the sagittal plane, and PSR is designed according to the planning (Figure 4). When a coronal deformity is also present, the calibrated anteroposterior full-spine X-ray will help to anticipate the length of PSR according to the amount of coronal correction planned. Once the planning is achieved, the surgeon will validate the design of PSR and can therefore obtain a pair of rods specifically for each patient.

We report here the feasibility and preliminary results of our experience with the use of PSR in the management of ASD about 77 cases. Parameters of the SRS-Schwab classification were measured preoperatively and at 3 months of follow-up (sagittal vertical axis (SVA), pelvic incidence and lumbar lordosis (PI-LL) mismatch, and pelvic tilt (PT)). Pre- and postoperative measurements were compared using a Student test (level of significance 5%).

## 3. Results

On the whole series, all surgeries were planned as described and all PSRs were used without further rod bending. Surgical indications included 43 ASD cases, 24 adolescent idiopathic scoliosis cases, and 10 deformity cases related to Parkinson's disease. Mean age was 59.1 years old ±18.6, and 71% of the patients were women.

**Figure 5:**
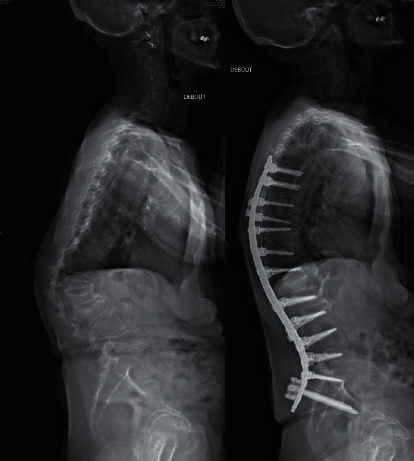
Example of sagittal realignment procedure using PSR in a patient with fixed thoracolumbar junction kyphosis (a). Surgical planning included a T6-ileum fixation with a L1 osteotomy and 3-month postoperative result (b).

Based on 3-month postoperative evaluation (Table 1), a significant decrease of SVA (−41%, *p* < 0.0001) and PI-LL (−62%, *p* < 0.0001) was reported. PT was not significantly corrected (−7%, *p*=0.154) (Figure 5).

For patients diagnosed with AIS, 15 levels were fused on average and a pedicle subtraction osteotomy (PSO) was done in 4 cases (17%). This subgroup of patients were aligned preoperatively and remained aligned postoperatively (postoperative SVA = 27 mm, PI-LL = 5°, and PT = 20°). Four patients had a preoperative hypokyphosis (<20°) and were corrected postoperatively.

For patients diagnosed with ASD, 14 levels were fused on average and a PSO was done in 12 cases (28%). A significant correction of SVA (77 vs. 42 mm, *p* < 0.001) and PI-LL (21 vs. 8°, *p* < 0.001) was noted. Pelvic tilt was not significantly modified (29 vs. 27°, *p*=0.437).

For patients diagnosed with Parkinson's disease, 17 levels were fused on average and a PSO was done in 7 cases (70%). In this subgroup of patients, measurements revealed a significant correction of SVA (100 vs. 47 mm, *p*=0.005) and PI-LL (29 vs. 6°, *p* < 0.001) but also of PT (32 vs. 25°, *p* < 0.001).

No intraoperative complications were reported; however, during the follow-up period, 7 patients (9%) presented a postoperative infection that required a revision surgery without implant removal and adapted antibiotic treatment. Mechanical complication rate at 3 months was reported at 4% (3 patients with proximal junctional kyphosis).

## 4. Discussion

Results from this preliminary study support the use of PSR for different reasons. Firstly, there is a crucial impact of preoperative surgical planning in order to reach postoperative alignment objectives. As reported by El Rahal et al. [7], risk for adjacent failures can be related to a lack of surgical planning or a problem during execution of the surgical procedure. This planning will allow simulations of various postoperative sagittal alignments according different types of surgery. It can also help to understand compensatory mechanisms related to the deformity and those that can occur after surgical correction and therefore help to establish the theoretical most appropriate surgical procedure taking into account Schwab's classification, Roussouly's types, theoretical lumbar lordosis, and age of the patient [7].

Secondly, with regards to sagittal correction, PSR can be a helpful tool in order to reduce the rate of undercorrected patients postoperatively, related to the lack of planning or suboptimal bending of rods during surgery [4]. The results presented here are in line with the study of Barton et al. [8] who also reported significant improvement of sagittal parameters using PSR. More recently, Solla et al. [9] reported their experience about 60 patients managed using PSR with a correction of PI-LL mismatch in 66% of the cases, a correction of SVA in 38% of the cases, and a correction of PT in 29% of the cases. According to their results, postoperative PI-LL mismatch was correlated with preoperative PI-LL and performing a PSO; postoperative SVA was correlated with preoperative SVA, performing a PSO, and age of the patient, and postoperative PT was correlated with preoperative PT. With regards to Parkinson's disease patients, a better correction was reported, especially for pelvic tilt. One reason for these findings might be related to the importance of initial deformity. In this subgroup of patients, preoperative malalignment was more important than in other groups, and as a consequence, a PSO was performed more frequently (70% of the cases) leading to a better restoration of lumbar lordosis and pelvic tilt [10].

With regards to AIS patients, a recent study by Solla et al. [11] reported the results of 37 patients managed using PSR. Their strategy involved an over bending of the rod in the concavity of the deformity which led to a correction of thoracic kyphosis for hypokyphotic patients underlining the interest of PSR in the management of AIS.

Finally, results from our study revealed a low rate of mechanical complications (4%) without rod breakage which is comparable with previous report [9]; however, our mechanical results must be taken into account cautiously due to the short follow-up (3 months).

Of course, this study presents also limits related to its design, the short-term follow-up, and the lack of clinical scores. It is also important to note that an initial learning curve is needed in order to plan and anticipate the surgical procedure in order to obtain PSR that will not require further modifications. When PSR is used, the strategy is to use the rods as a template for correction of the spine and not to adapt the rods to the spine. This point seems particularly important in our experience as it pushes the surgeon to follow the preoperative planning and avoid intraoperative changes (levels of fusion or osteotomy) that may actually be necessary in order to obtain optimal sagittal realignment.

While further studies will be needed in order to confirm these preliminary results, the use of PSR can be a turning point in the management of ASD. The exact knowledge of rod shape preoperatively will allow further postoperative analyses. It will therefore be possible to measure precisely the flattening effect of the rods, to anticipate further compensatory mechanisms in ASD and may be a way to decrease postoperative mechanical complications (proximal junctional failure or rod breakage).

## 5. Conclusion

Results from this preliminary study confirm the clinical feasibility of the use of PSR in the management of ASD. Three-month results are encouraging with regards to sagittal alignment. PSR can be valuable tools in order to optimize postoperative radiographic results even if further studies will be needed in order to confirm these results.

## Figures and Tables

**Table 1:** Radiographic results.

		Total (*n* = 77)	ASD (*n* = 43)	AIS (*n* = 24)	Parkinson (*n* = 10)
Age		59.1	66.7	42.0	68.1
Sex		*F* = 71%	*F* = 72%	*F* = 83%	*F* = 40%
Number of levels fused		14.5 ± 3.0	14.0 ± 3.4	14.8 ± 2.3	16.6 ± 1.3
PSO		24 PSO (31%)	12 PSO (28%)	4 PSO (17%)	7 PSO (70%)

SVA (mm)	Baseline	65.6 ± 65.7	77.3 ± 60.6	27.2 ± 58.0	100.3 ± 68.3
	3M	38.5 ± 36.6	41.9 ± 38.0	27.3 ± 32.1	47.3 ± 38.5
	∆ (*p* value)	−41% (*p* < 0.0001)	−46% (*p* < 0.0001)	0% (*p*=0.997)	−53% (*p*=0.005)

PI-LL (°)	Baseline	18.1 ± 20.3	20.8 ± 17.8	7.8 ± 23.7	28.8 ± 10.9
	3M	6.9 ± 11.8	8.3 ± 12.8	5 ± 10.9	5.5 ± 10.3
	∆ (*p* value)	−62% (*p* < 0.0001)	−60% (*p* < 0.0001)	−35% (*p*=0.458)	−81% (*p* < 0.0001)

PT (°)	Baseline	26.3 ± 11.3	28.6 ± 9.9	19.7 ± 11.8	31.7 ± 9.9
	3M	24.6 ± 11.9	27.1 ± 12.9	20.4 ± 11.0	24.5 ± 6.7
	∆ (*p* value)	−7% (*p*=0.154)	−5% (*p*=0.437)	+4% (*p*=0.663)	−23% (*p* < 0.001)

## Data Availability

Data related to the study are available on request.
